# Supplementary material for: Clinical Experience with Genome-Wide Noninvasive Prenatal Screening in a Large Cohort of Twin Pregnancies
Source: Genes (Basel). 2023 Apr 26;14(5):982. doi: 10.3390/genes14050982 (PMC10218036; doi:10.3390/genes14050982)
Supplement: Supplementary file 1 [file genes-14-00982-s001.zip › genes-2341087-supplementary.pdf]

Table S1: NIPT performance for detecting trisomies 21, 18, 13 in 8 triplet pregnancies

	Maternal Age	Gestational Age (weeks)	Method of conception	FF (%)	Chorionicity	NIPT results
1	41.94	13+3	spontaneous	12	Monochorionic	Negative
2	38.67	13+5	art	15	Trichorionic	T21 (confirmed with amniocentesis)
3	32.55	13	art	15	Trichorionic	Negative
4	37.02	11	spontaneous	15	Trichorionic	Negative
5	35.15	10+4	spontaneous	10	Trichorionic	Negative
6	43.82	11+5	art	18	Trichorionic	Negative
7	39.13	13+4	art	12	Trichorionic	Negative
8	28.18	13+1	art	10	Trichorionic	Negative
9	30.78	13+2	spontaneous	19	Trichorionic	Negative
10	50.68	11+4	art (one embryo transferred)	8	Monochorionic	Negative

art, assisted reproductive technology
